# IMACULAT — An Open Access Package for the Quantitative Analysis of Chromosome Localization in the Nucleus

**DOI:** 10.1371/journal.pone.0061386

**Published:** 2013-04-08

**Authors:** Ishita Mehta, Sandeep Chakraborty, Basuthkar J. Rao

**Affiliations:** 1 Department of Biological Sciences, Tata Institute of Fundamental Research, Mumbai, India; 2 Department of Biological Sciences, Tata Institute of Fundamental Research, Mumbai, India; 3 Department of Biological Sciences, Tata Institute of Fundamental Research, Mumbai, India; University of Minnesota, United States of America

## Abstract

The alteration in the location of the chromosomes within the nucleus upon action of internal or external stimuli has been implicated in altering genome function. The effect of stimuli at a whole genome level is studied by using two-dimensional fluorescence in situ hybridization (FISH) to delineate whole chromosome territories within a cell nucleus, followed by a quantitative analysis of the spatial distribution of the chromosome. However, to the best of our knowledge, open access software capable of quantifying spatial distribution of whole chromosomes within cell nucleus is not available. In the current work, we present a software package that computes localization of whole chromosomes - Image Analysis of Chromosomes for computing localization (IMACULAT). We partition the nucleus into concentric elliptical compartments of equal area and the variance in the quantity of any chromosome in these shells is used to determine its localization in the nucleus. The images are pre-processed to remove the smudges outside the cell boundary. Automation allows high throughput analysis for deriving statistics. Proliferating normal human dermal fibroblasts were subjected to standard a two-dimensional FISH to delineate territories for all human chromosomes. Approximately 100 images from each chromosome were analyzed using IMACULAT. The analysis corroborated that these chromosome territories have non-random gene density based organization within the interphase nuclei of human fibroblasts. The ImageMagick Perl API has been used for pre-processing the images. The source code is made available at www.sanchak.com/imaculat.html.

## Introduction

DNA is folded and compacted in order to occupy the limited space within the cell nucleus wherein the genome replicates, transcribes and translates to form proteins [1]. Another important revelation in the field of genomics came from the finding that the genomes are positioned in a non-random manner in the cell nucleus [2]–[5]. The organized genome and the machineries required for its maintenance and function within the nucleus, along with other nuclear components make up the nuclear architecture [6]–[8]. The organization of the genome within the nucleus and its interaction with nuclear components alter during both development and in disease. This non-random chromosomal positioning within the architectural framework of the nucleus is thought to be a critical dimension of genome function [5], [8]–[11].

Chromosome territories occupy a non-random and a radial distribution within interphase nuclei [2]–[5], [10], [12], [13]. Although genomic entities are arranged in this patterned organization, they are not rigid compartments but instead are dynamic structures that can be repositioned with respect to other nuclear structures and other genomic regions [14]. In addition, dynamic repositioning of whole chromosome territories has also been observed during differentiation [8], [15]–[19] and when cells exit the proliferative cell cycle to become quiescent or senescent [20], [21].

Positions of chromosome territories can be delineated using *in situ* hybridization (FISH) techniques employing whole chromosome probes [22]–[24]. Although, three-dimensional FISH assures accurate localization measurements, this technique is time consuming [23], [25]. It is thus very difficult to use this technique while looking at global alterations in spatial locations of large number of chromosomes. An easier alternative to this is to perform FISH on completely flattened nuclei (2D-FISH) [2], [5]. The ease of this technique and higher probe penetration of flattened nuclei assists in performing large number of hybridizations at once. In addition image capturing is easier in case of 2D-FISH, thus allowing larger numbers of nuclei to be captured. However, one of the major hurdles in performing 2D-FISH is the lack of open access software that could be used to quantify the localization of chromosomal territories.

Previously, a free open-source image analysis tool (IMAJIN COLOC) was developed to do multiple Z plane images (Z stacks) [26]. Another co-localization method has proven to be robust in recognizing co-localizations in the presence of background noise [27]. Several commercial packages for doing Z stack analysis are also available - Imaris (http://www.bitplane.com/go/products/imaris) and the Zeiss LSM software (http://microscopy.zeiss.com/microscopy/en_de/home.html). However, these are not suited for 2D-analysis. One such application from IPLab Spectrum software (http://www.spectraservices.com/IPLAB.html) that has been used for similar 2D-analyses [2], [5], [21], has now discontinued the development of the product. Further, an open source package provides a user with the opportunity to fine tune the package according to their requirements.

In the current work, we present software that computes localization of whole chromosomes - Image Analysis of Chromosomes for computing localization (IMACULAT). We partition the cell into concentric elliptical compartments of equal areas, and the variance in the quantity of any chromosome is used to determine its movement in the cell. The results are outputted to a text file, and a corresponding gif image showing the elliptical shells is generated for visual comparison with the original image. Automation allows high throughput analysis for deriving statistics that are used to validate a hypothesis regarding the position of any chromosome. The ImageMagick Perl API libraries (http://www.imagemagick.org/) have been used for pre-processing the images.

In order to validate the functionality of IMACULAT, we have mapped the positions of all human chromosomes in normal human dermal fibroblasts. The locations of these human chromosomes corroborated earlier published studies [2], [5], [21]. Additionally, in concurrence with previous reports, we observed a gene density based organization of chromosomes with gene-rich chromosomes (19, 17, etc.) occupying the center of the nucleus while gene-poor chromosomes (18, 2, etc.) localizing at the nuclear periphery. Thus, this validation establishes IMACULAT as an automated quantitative methodology that can be routinely used to map positions of components within the nucleus.

## Results and Discussion

Understanding the genome and its function is vital in the field of biomedical research. The human genome sequencing project has laid the foundation towards this goal whereby the sequence of 3 billion bases of human DNA was determined and approximately 30,000–40,000 protein coding genes in the human genome have been identified [28], [29]. However, it is important to remember that genomes are not a single dimensional entity and elucidation of DNA sequence was only the starting point of genomics research. In reality, it is vital to extrapolate the DNA sequence information from the human genome project onto genome function, which is the major goal of the post-genomic era.

Chromosomes throughout most of the cell's life span occupy a distinct three-dimensional location within the interphase nuclei, which are known as chromosome territories [5], [21]. Since cells spend most of their life span in interphase and also most biological activities occur during this phase of the cell cycle, it is important to understand the dynamics of the interphase genome [30]. Spatial organization of chromosomes is thought to affect various important biological processes such as transcription, replication as well as cellular differentiation [8], [15], [31]–[35].

Proliferating normal human dermal fibroblasts (NHDFs) were subjected to a standard two-dimensional fluorescence *in situ* hybridization (FISH) to delineate territories for all human chromosomes (22 autosomes, X and Y). A non-random distribution was observed for all human chromosomes in interphase nuclei, and with each chromosome occupying a specific location [2], [5], [21]. For instance, territories of chromosome 19 are enriched at the center of the nuclei while those of chromosome 12 are known to localize at the nuclear periphery [5], [21]. On the other hand, chromosome 8 has an intermediate position within interphase nuclei [5], [21]. These spatial distributions of chromosomes within the nuclei have been known to affect genome functions [8], [36]–[38].

**Figure 1:**
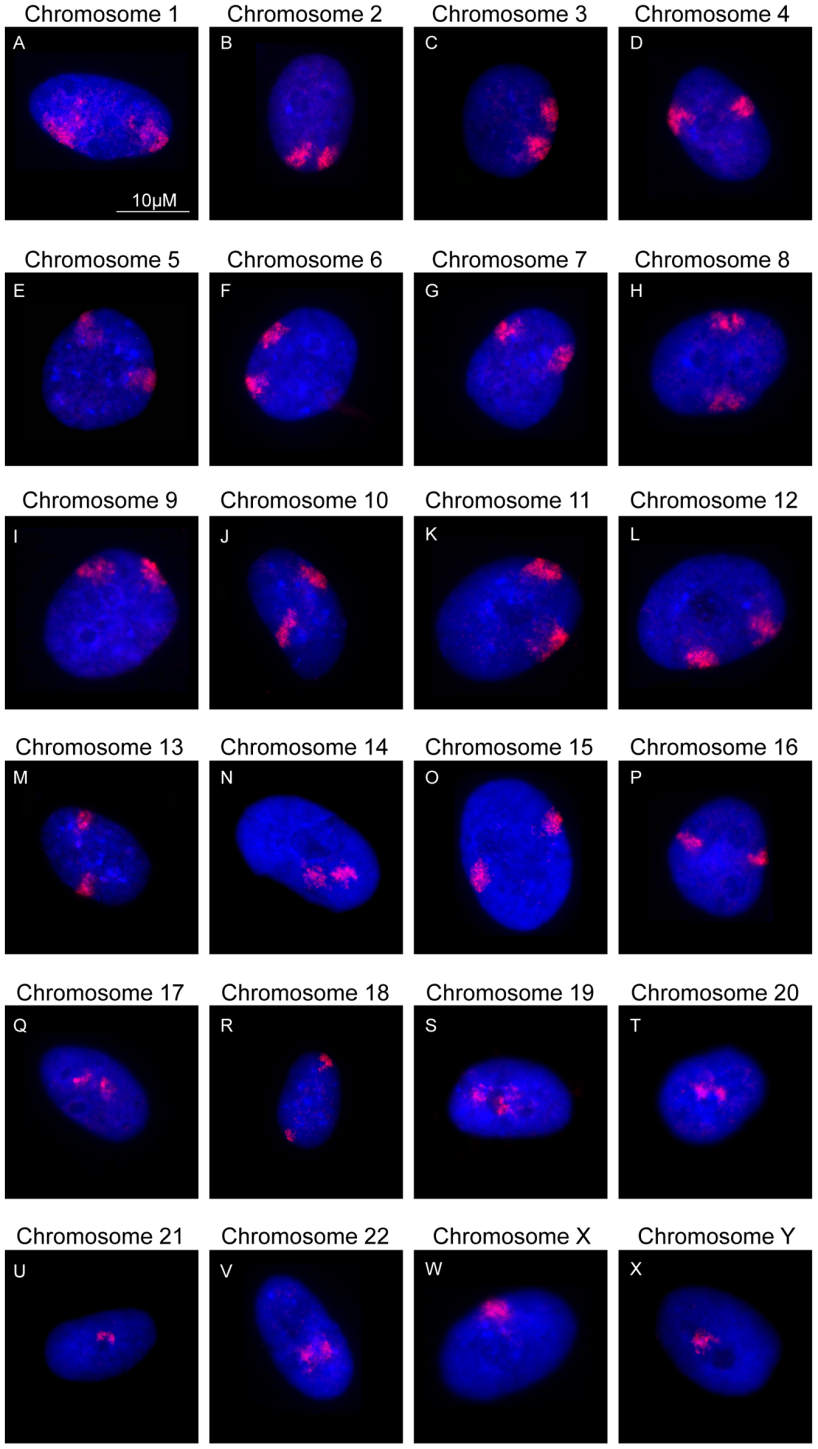
Positions of all chromosomes in normal proliferating human dermal fibroblasts: Images displaying the spatial arrangement of each of the human chromosome territories (in red) in interphase nuclei (stained in blue) of fibroblasts. The numbers on the top of each nucleus indicates the chromosome to which a specific probe was hybridized to, as revealed by FISH. Scale bar = 10 µM.

**Figure 2:**
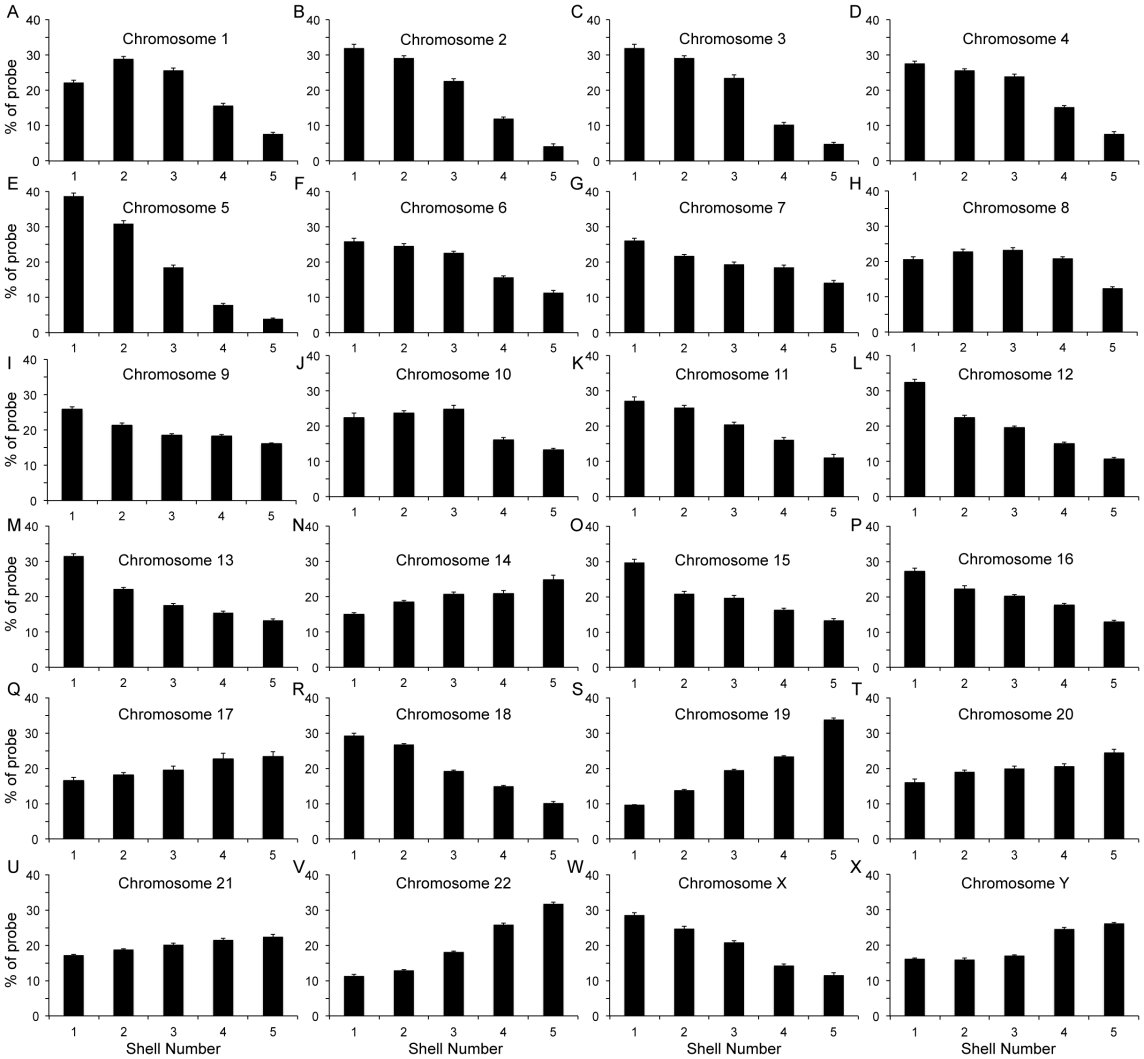
Histograms plotted from output of IMACULAT displaying positions of all human chromosomes in normal proliferating human dermal fibroblasts: Normal human dermal fibroblasts were subjected to standard 2D-FISH assay. At least 100 digital images were analyzed per chromosome by IMACULAT. The graphs display the % probe intensity of each human chromo-some in each of the shells (y-axis), and the shell number on the x-axis. The standard error bars representing the standard errors of mean (SEM) were plotted for each shell for each graph.

Using IMACULAT, we positioned the territories of all human chromosomes within interphase nuclei of normal proliferating human dermal fibroblasts. Representative images and the output of IMACULAT as histograms for each chromosome have been displayed in Fig 1 and 2 respectively. Each nucleus was divided into 5 concentric shells of equal area and the amount of probe corresponding to the chromosome signal, in each shell was quantified (See methods). We observed that % probe signal for most gene-rich chromosomes, for example chromosomes 17 and 19 (Fig 2 panels Q and S, respectively) were enriched in the interior-most shell (Shell 5) and subsequently lowered with decreasing shell number. Thus, the positive slope on the histogram corroborates the interior localization of these chromosome territories (Fig 2 panels Q and S). Similarly, for gene-poor chromosomes, such as chromosomes 13 and 18, the innermost shell (Shell 5) showed the least % probe signal, which increased with radial distance, the signal being the maximum in the outermost shell (Shell 1) (Fig 2 panels M and R, respectively). Hence, the negative slope indicated the already known peripheral location of chromosomes 13 and 18 territories (Fig 2 panels Q and S, respectively). Finally, bell shaped curves of chromosomes, such as chromosome 8, was indicative of its intermediate location within an interphase nucleus (Fig 2 panel H). IMACULAT output for three chromosomes can be accessed at http://www.sanchak.com/imaculat/sampleruns/.

Using IMACULAT, we have corroborated an already existing correlation between gene-density and non-randomness of chromosomal location within an interphase nucleus. For example, the gene-dense chromosome 19 is known to localize at the nuclear interior while nuclear periphery is enriched with gene-poor chromosomes such as chromosome 18 [5], [21]. Studies have indicated a relationship between chromosome location and genome function, whereby transcription is enriched at the nuclear center as compared to the periphery [15], [31]–[35]. In corroboration with this, euchromatin is enriched at the nuclear interior while most heterochromatin is localized at a peripheral location within the nucleus [8], [39]–[42]. Spatial distribution within the nucleus is also known to affect other genome functions such as DNA repair [43]–[47] and replication [48]–[51]. While a 3D approach to the positioning of chromosomes is increasingly gaining precedence it has been noted that the `2D approach is still the most commonly used and will likely remain the most relevant for a considerably long time' [52]. In the current work, IMACULAT recapitulated that all human chromosome territories occupy a non-random location within the interphase nuclei of human fibroblasts, and thus provides an invaluable computational tool to quantify such spatial distributions.

## Materials and Methods

**Figure 3:**
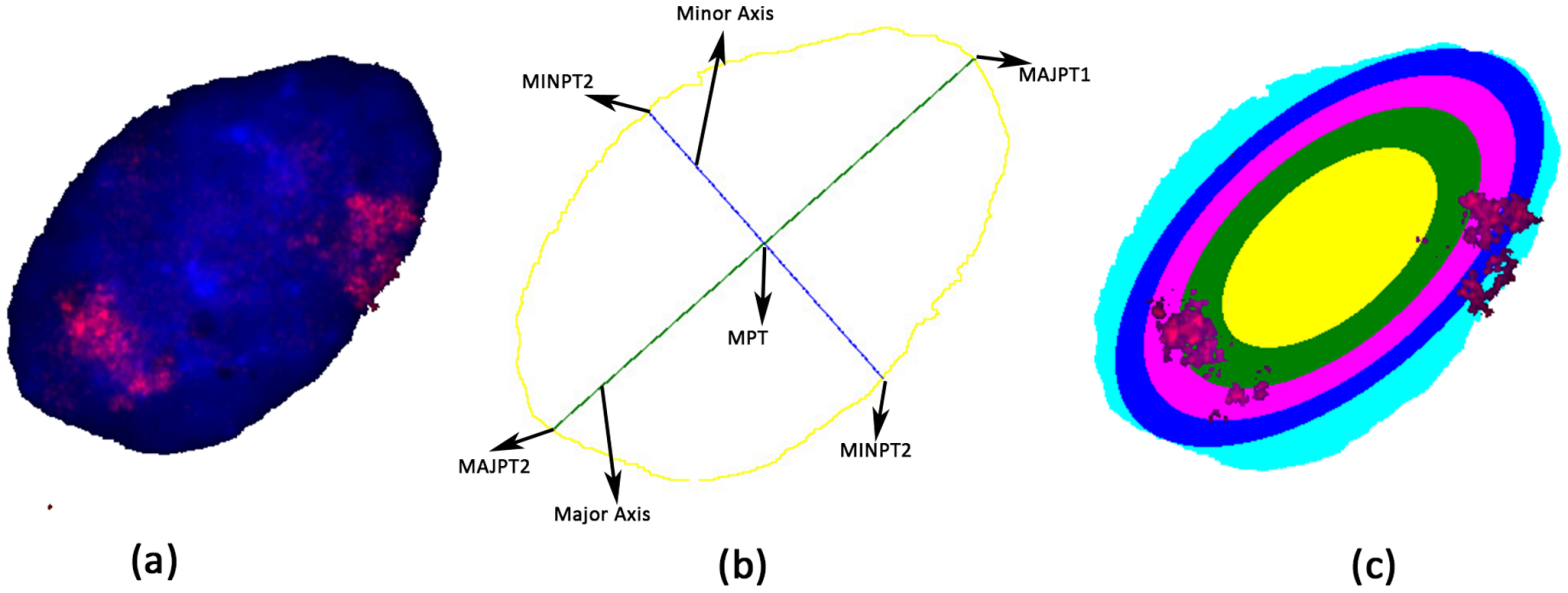
Steps in quantifying chromosome localization: (a) The original image obtained from two-dimensional fluorescence in situ hybridization (FISH). The background, cytoplasm and chromosome are colored white, blue and red respectively. (b) The contour is colored yellow, while the major and minor axes are in green and blue respectively. (c) The nucleus is partitioned into five concentric shells of equal area, and the percentages of chromosome in each shell are computed using the number of red pixels (Table 1).

The input to IMACULAT is a rectangular image of an elliptical nucleus which is separated into the background, the cytoplasm and the labeled chromosome(s), each of which has a parameterized color (white, blue and red respectively in the examples presented here) (Fig. 3a). The ImageMagick package parses this image into a grid of pixels (each pixel is represented by the triplet RGB values) of height `H' and width `W' (W = 401, H = 385 for Fig. 3).

### 3.1 Identifying the contour of the nucleus

The first step is to identify the contour of the nucleus. The underlying idea is to scan from the right (or left) from the point x = 1 (x = W for scanning from the left), till we encounter the first nuclear pixel (blue) or chromosome (red), for every height y = 1 to y = H. The contour is thus defined by two columns of height `H' - LCountour and RCountour - such that either LCountour[i] = RCountour[i] = 0 (there is no part of the nucleus in this horizontal line), or LCountour[i]< = RCountour[i]. The contour is colored yellow in Fig. 3b.

### 3.2 Determining the major and minor axes

One cannot make any assumption of the orientation of the nucleus. First, we determine the bounding box coordinates of the nucleus from the contour–top_x,y_, bottom_x,y_, right_x,y_, left_x,y_. The mid point of the nucleus (MPT) is computed as:

MPT_x_ = (right_x_+left_x_)/2; MPT_y_ = (top_y_+bottom_y_)/2;

The intersection of the major axis to the perimeter is computed as the point (from all the contour points), which has the maximum distance from MPT. Let us denote it as MAJPT1. The line connecting MAJPT1 and MPT is extended till it hits the opposite side of the perimeter, and defines the other end of the major axis (MAJPT2). Since the nucleus is rarely a perfect ellipse, we recalculate the midpoint MPT as:

MPT_x_ = (MAJPT1_x_+MAJPT2_x_)/2; MPT_y_ = (MAJPT1_y_+MAJPT2_y_)

The minor axis is computed by extending a line perpendicular from MPT, such that it makes two intersections with the perimeter (MINPT1 and MINPT2). The major and minor axes are shown in Fig. 3b in green and blue respectively.

### 3.3 Partitioning the nucleus into concentric shells

Let us denote the length of the major and the minor axis as `2A' and `2B' respectively. The area of the ellipse is defined as - AREA = π*A*B. We now proceed to divide the nucleus into N concentric ellipses (five in this case, Fig. 3c). The innermost ellipse should have an area of AREA*1/N, the next ellipse should have area of AREA*2/N and so on. This results in the innermost shell (colored yellow in Fig. 3c) having an area of AREA*1/N, the next shell (colored green in Fig. 3c) with an area of (AREA*2/N–AREA*1/N = AREA*1 = 1/N) and so on. The last shell (colored aqua in Fig. 3c) has by the preceding logic an area of AREA*1/N), although this shell has an irregular shape. Such a bottom-up approach in identifying the shell obviates erosion analysis to smoothen the nuclear periphery. In fact, our analysis is truer in the sense that erosion analysis makes modifications to increase depressed regions and suppress protrusions, although on an average they are expected to offset each other. Another constraint is that for each of the concentric ellipses, the ratio between the major and minor axis (A = B) is to be maintained.

Table 1 shows the number of pixels within each shell, and the distribution of cytoplasm, chromosome and unidentified colors. The colors are identified within a range of values for RGB. For example, `pure' red has the value [1, 0, 0], but a value of [0.9, 0.1, 0.1] is also considered as being red. It can be seen that the unidentified colors are negligible.

**Table 1:** Partitioning of the nucleus into concentric ellipses: The areas are computed by the number of pixels as shown in Fig. 2c.

Shell Number	Color	Blue	Red	Others	All	% Blue	% Red	% Others
1	Yellow	12207	10	0	12217	99.9	0.1	0
2	Green	10936	1192	0	12128	90.2	9.8	0
3	Magenta	11015	1221	0	12236	90	10	0
4	Blue	11483	716	5	12204	94.1	5.9	0
5	Aqua	11406	653	34	12093	94.3	5	0.3

The cytoplasm and labeled chromosome are colored blue and red respectively. Any color not recognized as either of these two colors is specified as `Others'. Shells are numbered from the periphery inwards.

### 3.4 Removing smudges outside the nucleus

**Figure 4:**
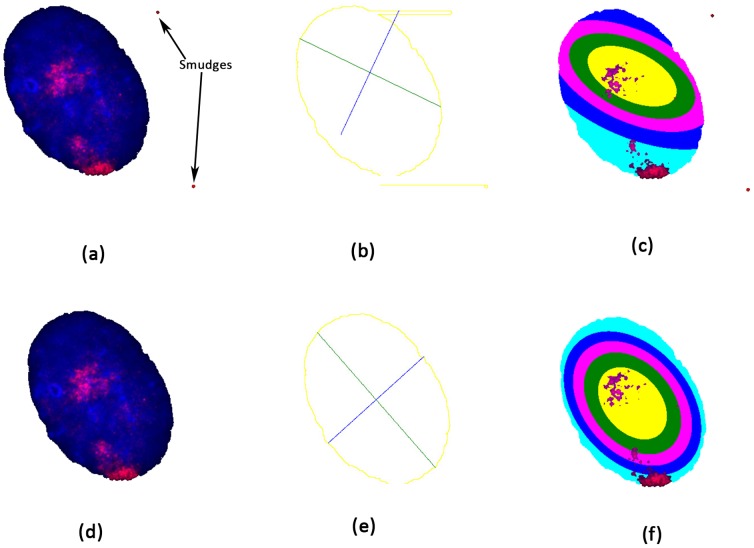
Steps in removing smudges outside the nucleus boundary: (a) There are a couple of unwanted smudges outside the nucleus boundary colored red. (b) These smudges pose a problem while computing the contour, which assumes the presence of any color other than background color (white) as the beginning of the nucleus. The major and minor axes can be seen to be erroneous. (c) Consequently, the partitioning of the nucleus is incorrect too. (d) The pre-processing step removes the smudges. See Methods section for the detailed algorithm. (e) The computation of the contour, the major and the minor axis are now correct. (f) The erroneous partitioning is thus fixed.

Occasionally, we encounter images in which there are smudges outside the nucleus boundary (Fig. 4a). These colored portions pose a problem to the algorithm, which determines the contour, and the major/minor axes of the nucleus (Fig. 4b), and consequently the nucleus partitioning (Fig. 4c). We introduce a pre-processing step to remove these smudges, which results in a representation of the nucleus that corrects the erroneous calculations (Figs. 4 d, e and f). The underlying concept in identifying a color outside the nucleus is to determine the circumference of a specified radius (five pixels in the current examples), and ensure that the circumference has the background color (white in this case). There are two possible cases that will escape this identification algorithm. The first is when the smudge is very close to the nucleus (within the specified radius), and thus should not introduce any significant error if left undetected. The second case occurs when the smudge is larger than the specified radius. Such images are rare, and are removed by visual inspection.

### 3.5 Installation, running the program and analyzing the results

The IMACULAT package is written in Perl on Ubuntu. Hardware requirements are modest - all results presented here are from a simple workstation (8GB ram) and runtimes per image were a few Minutes, at the most. The source code and manual are made available at www.sanchak.com/imaculat.html. The installation requires the following supplementary packages - ImageMagick (available at http://www.imagemagick.org/) and the Perl api package (Image::Magick), which can be obtained from www.cpan.org.

In order to simplify running the program, we have created a wrapper C-shell script that takes two parameters - a file containing the name of the image files to be processed, and the name of the results directory. The output is an Excel (.xls) sheet, which details the % of the probe present in each shell. Other outputs show the identified colors in the images as intermediate files. We have provided three sample directories, which contain the original images and the IMACULAT results, at www.sanchak.com/imaculat/sampleruns.tgz. They are also present in a directory structure for easy browsing at www.sanchak.com/imaculat/sampleruns/.

### 3.6 In *vitro* methods

Proliferating normal human dermal fibroblasts (NHDFs) [Lonza] were maintained in 15% FBS-DMEM. Spatial locations of chromosomes were delineated using standard two-dimensional fluorescence *in situ* hybridization protocol [2]. Briefly, cells were trypsinized, treated with hypotonic solution and fixed with methanol:acetic acid (3∶1). Further, cells were taken through an ethanol row followed by denaturation using 70% (v/v) formamide at 70 °C and hybridization with whole chromosome probes [Applied Spectral Imaging]. The slides were then washed and mounted in Vectashield mounting media containing DAPI [Vectashield]. At least 100 images were captured per chromosome using Zeiss Axiovert 200 microscope (Axiovision software). Spatial positions of chromosome territories in these images were obtained by running them through IMACULAT. Histograms displaying these results and standard error bars representing the +/− standard error of mean (SEM) were plotted.
